# Energy Balance of Wireless Sensor Nodes Based on Bluetooth Low Energy and Thermoelectric Energy Harvesting

**DOI:** 10.3390/s23031480

**Published:** 2023-01-28

**Authors:** Yuming Liu, Jordi-Roger Riba, Manuel Moreno-Eguilaz

**Affiliations:** 1 Electrical and Electronics Engineering Departments, Universitat Politècnica de Catalunya, Rambla Sant Nebridi 22, 08222 Terrassa, Spain; 2 SBI Connectors, Sant Esteve Sesrovires, Albert Einstein, 5, 08635 Sant Esteve Sesrovires, Spain

**Keywords:** energy harvesting, thermoelectric generator, high voltage, substation connector, battery efficiency, power consumption

## Abstract

The internet of things (IoT) makes it possible to measure physical variables and acquire data in places that were impossible a few years ago, such as transmission lines and electrical substations. Monitoring and fault diagnosis strategies can then be applied. A battery or an energy harvesting system charging a rechargeable battery typically powers IoT devices. The energy harvesting unit and rechargeable battery supply the sensors and wireless communications modules. Therefore, the energy harvesting unit must be correctly sized to optimize the availability and reliability of IoT devices. This paper applies a power balance of the entire IoT device, including the energy harvesting module that includes two thermoelectric generators and a DC–DC converter, the battery, and the sensors and communication modules. Due to the small currents typical of the different communication phases and their fast-switching nature, it is not trivial to measure the energy in each phase, requiring very specific instrumentation. This work shows that using conventional instrumentation it is possible to measure the energy involved in the different modes of communication. A detailed energy balance of the battery is also carried out during charge and discharge cycles, as well as communication modes, from which the maximum allowable data transfer rate is determined. The approach presented here can be generalized to many other smart grid IoT devices.

## 1. Introduction

Wireless sensor nodes (WSNs) enable connecting things to the internet through a gateway interfacing the internet and the WSNs. WSNs allow collecting sensed data and send this information to the gateway using a one-way or two-way communication protocol [1]. Internet of things (IoT) devices allow the monitoring of different physical objects [2,3] while enabling real-time health condition approaches to be applied. It is known that electrical connections are among the critical points in power systems, often being placed in remote or inaccessible locations, so they deserve special attention [4]. It is interesting to provide the connections with the ability to measure fundamental physical variables, as well as to communicate, in order to determine their state of health and remaining useful life (RUL) [5,6]. With the development of IoT technology, today this goal is within reach. Devices designed for this purpose must include an energy harvesting unit, specific sensors, and a communication module to acquire the key physical variables and send this information wirelessly to the cloud to be analyzed in real time to apply predictive maintenance approaches [7]. The required energy and communication capabilities are critical factors, which are highly influenced by factors such as data transfer rate and distances to be covered [8]. In any case, the energy harvesting unit plays a key role. The energy harvesting unit converts the energy from the environment into electrical energy [9], being the unit in charge of supplying the required energy to the IoT system at the right time. Energy harvesters also enable prolonging the life of battery-powered WSNs, as they allow the battery to be recharged [10] and minimize maintenance costs [11].

Transmission systems are a fundamental part of today’s electrical grid. To ensure stable and reliable power grid operation, key parameters of transmission systems need to be measured, monitored, and analyzed in real time. They can operate in both direct and alternating current power systems. WSNs have a key role in meeting this need, since they are the devices in charge of measuring basic data and sending this information to the gateway. Based on the analysis of this information, predictive maintenance approaches can be applied to optimize the reliability, availability, and stability of the electrical grid. This strategy also makes it possible to minimize human intervention in the data acquisition process, which is especially important in remote and difficult-to-access areas [4], where human intervention can be very expensive and even unsafe. Therefore, self-powered WSNs can be very useful for monitoring transmission systems [12].

This paper performs an energy balance of the *SmartConnector*, an IoT device that includes a thermoelectric energy harvesting system, an energy storage unit, and different electronic sensors (current, voltage drop and temperature) to estimate the electrical contact resistance of the connector, a microprocessor, and a communications module. There is a shortage of works that perform an energy balance of the full system, so this work contributes to this area. The *SmartConnector* is a challenging project because these electronic modules must be added to aluminum substation connectors, which operate outdoors at voltage levels up to 550 kV. The *SmartConnector* can measure in situ and in real-time different parameters of the high-voltage substation connector, which can be used to determine the state of health or the remaining useful life. The data are transmitted wirelessly to a nearby gateway, which sends the data to the cloud for further visualization and analysis.

**Figure 1:**
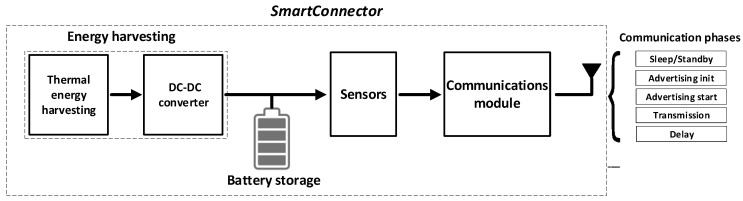
Diagram of the wireless *SmartConnector* device.

Figure 1 schematizes the main modules of an IoT-WSN for substation connectors, here called *SmartConnector*. Due to the limited amount that the energy harvesting unit can generate, WSs usually operate in an intermittent on-off pattern [4,13], so communication protocols typically have different phases. The energy harvesting unit analyzed in this paper is based on a solid-state thermoelectric generator (TEG), which transforms a temperature difference into useful electrical energy.

This paper applies an energy balance of the entire *SmartConnector* device, including the energy harvesting module, DC–DC converter, battery and sensors, and communication modules. Due to the small currents that intervene in the different phases of the communications and the fast-switching pulses that characterize these currents, special care must be taken when measuring the energy in each phase. Therefore, very specific and expensive instrumentation is required. This paper shows that using conventional instrumentation it is also possible to measure the energy involved in each phase of the communications. Since the *SmartConnector* is installed on large tubular aluminum busbars, there is a small temperature gradient between the ambient and the busbar. This work also focuses on a challenging problem, the thermoelectric energy harvesting under very low temperature gradients, which has been poorly studied in the technical literature. Due to these unfavorable conditions, thermoelectric generators produce a very low voltage, which requires a suitable DC–DC converter. In addition, a detailed energy balance of the thermoelectric energy harvesting unit and the battery is also carried out during the charge and discharge cycles, as well as during the communication phases, this being a novelty of this work. The energy balance allows determining the maximum data transfer rate (DTR_max_), that is, the maximum communication cycles per hour the *SmartConnector* can do without draining the batteries. Although the results presented in this paper have been applied to a particular IoT device, the *SmartConnector*, the approach presented here can be generalized to many other smart grid IoT devices incorporating energy harvesting units, such as triboelectric or piezoelectric nanogenerators. These results could potentially contribute to improve the energy management and lifetime of WSNs.

## 2. System Efficiency

This section describes the system to determine the efficiency of the entire system comprising the energy harvesting system (TEGs + DC–DC converter), the battery, and the sensors and communication modules.

### 2.1. Energy Harvesting Efficiency

A thermoelectric generator (TEG) is used to capture energy from the thermal gradient existing between a substation busbar and the environment. Substation busbars, usually hollow cylindrical aluminum tubes, are common connection nodes for multiple incoming and outgoing circuits. Due to the low temperature gradient between the busbar and the environment, special care must be taken to select the most appropriate TEG. This unfavorable condition forces the TEG to generate a very low voltage, some fractions of a volt, requiring a suitable DC–DC converter to charge the battery and supply the sensors and the communications module. Therefore, the energy harvesting system consists of a TEG module and a DC–DC converter with a very low input voltage range.

The specific efficiencies of the TEG, *η_TEG_*, and of the DC–DC converter, *η _DC/DC_*, must be calculated to determine the efficiency of the entire energy harvesting system (TEG + DC–DC converter), *η_TEG+DC/DC_*.

The steady state heat transfer equation in a busbar can be expressed as [14],

(1) $$I_{RMS}^{2}r_{ac}(T)-p_{c}-p_{r}=0\;[W/m]$$

*I_RMS_* [A] being the electric current flowing in the busbar, *r_ac_*(*T*) [Ω/m] the per unit length ac resistance of the busbar at the operating temperature *T* [°C], and *p_c_* [W/m] and *p_r_* [W/m] being the cooling terms due to natural convection and radiation, respectively.

The resistance term *r_ac_* [Ω/m] can be measured as [5,15]

(2) $$r_{ac}(T)=\frac{\Delta V_{1m}}{I}\cos\varphi \;[\Omega /m]$$

where Δ*V*_1m_ [V] is the voltage drop measured between two points of the busbar separated by 1 m, *I* [A] is the ac current flowing through the busbar, φ [rad] is the phase shift between the voltage drop and the current, and *T* [°C] is the temperature of the busbar.

The efficiency of the TEG is calculated as

(3) $$\eta_{TEG}=\frac{P_{out,TEG}}{P_{inp,TEG}}=\frac{P_{electric,TEG}}{P_{Joule,TEG-area}}$$

where *P_electric,TEG_* [W] is the electric power generated by the TEG, and *P_Joule,TEG-area_* [W] is the Joule heat generated by the busbar in the area of the TEG (80 mm × 40 mm), which can be calculated as

(4) $$P_{Joule,TEG-area}=P_{Joule,conductor}\frac{A_{TEG}}{A_{Conductor}}\;[W]$$

where *A_TEG_* [m^2^] and *A_Conductor_* [m^2^] are, respectively, the area of the outer surfaces of the TEG and conductor, and *P_Joule,conductor_* [W] is the power loss in the busbar due to the Joule effect.

Finally, the energy efficiency of the DC–DC converter is calculated as the ratio between the output and input electrical powers as

(5) $$\eta_{DC-DC}=\frac{P_{out\_DC-DC}}{P_{inp\_DC-DC}}=\frac{V_{out}I_{out}}{V_{inp}I_{inp}}\;[-]$$

*V_out_*, *I_out_*, *V_inp_*, and *I_inp_* being the output and input voltages and currents of the DC–DC converter.

### 2.2. Battery Efficiency

Today, rechargeable batteries play a leading role in energy management for IoT applications. Various methods have been proposed to estimate battery lifetime and the state of health, which can be based on fast impedance measurements [16], or on health indicators based on the internal resistance because it is greatly impacted by ageing [17], or on the capacity level [18] among others.

It is known that, as a result of energy loss in battery operation, additional energy is required, so battery energy efficiency is a relevant factor of battery economy. Battery energy efficiency characterizes the utilization rate during energy conversion from chemical energy to electrical energy [19]. In order to minimize the energy losses in the batteries and to evaluate the energy efficiency of the entire thermal energy harvesting system, this paper analyzes the efficiency of the batteries under a very low current rate, since the analyzed application is characterized by very low current rates.

In [20] it is concluded that for nickel metal hydride (Ni-MH) batteries, full charge cannot be reached without overcharging due to side reactions. Ni-MH batteries are applied in many crucial applications such as wearable electronic devices and hybrid vehicles due to the high cycle life and robustness [10,21]. This paper analyzes a pack of two series connected Ni-MH batteries. It is important to determine the energy loss in the batteries and the input and output energies in the batteries during the charge and discharge cycles to characterize their efficiency and energy behavior.

Since the IoT device analyzed in this paper has a very low power consumption, battery efficiencies under low current rates are studied. These efficiencies have three components, that is, charge efficiency *ƞ_charge_*, discharge efficiency *ƞ_discharge_*, and overall efficiency *ƞ_Battery_*.

The energy efficiency under charging conditions [20] is the ratio between the chemical energy gained by the battery during the charge cycle Δ*E_Battery input_* [J] and the energy extracted from the power source Δ*E_Power source_* [J].

(6) $$\eta_{Charge}=\frac{\Delta E_{Battery\;input}}{\Delta E_{Power\;source}}=\frac{\Delta E_{Battery\;input}}{\Delta E_{Battery\;input}+\Delta E_{Chargingloss}}$$

where Δ*E_Charging loss_* [J] is the energy loss in the battery during the charging cycles due to Joule heating and electrochemical reaction processes [20,22]. Δ*E_Battery input_* is the chemical energy stored in the battery, i.e., the net energy. The recharged energy and the net energy are not the same because the recharged electric energy cannot be completely transformed into chemical energy [19].

The energy extracted from the power source, Δ*E_Power source_* [J], can be determined as [19]:

(7) $$\Delta E_{Power\;source}=\int_{t_{0}}^{t}V_{Charge}I_{Charge}dt=\int_{SoC(t_{0})}^{SoC(t)}V_{Charge}C_{n}dSoC\;[J]$$

The net energy gained by the battery during the charge cycle, Δ*E_Battery input_*, can be expressed as [19]

(8) $$\Delta E_{Batttery\;input}=\int_{SoC(0)}^{SoC(t)}V_{OCV}(SoC)C_{n}dSoC\;[J]$$

where *SoC(t_0_)* [-] is the initial state of charge, *SoC(t)* [-] is the final state of charge,*V_Charge_* [V], and *I_Charge_* [A] are the battery voltage and current during the charge process, respectively, *V_OCV_* [V] is the open circuit voltage, and *C_n_* [Ah] is the rated capacity of the battery.

The state of charge (*SoC*) of the battery can be calculated as [19]:

(9) $$SoC(t)=SoC(t_{0})+\frac{1}{C_{n}}\int_{t_{0}}^{t}I_{Charge}dt\;\mathrm{or}\;SoC(t)=SoC(t_{0})-\frac{1}{C_{n}}\int_{t_{0}}^{t}I_{discharge}dt\;$$

The rated capacity *C_n_* [Ah] of the battery plays a major role in calculating the net energy Δ*E_net_* and in determining the *SoC*. The method for determining *C_n_* is described in Section 4.2.

The energy efficiency under discharge conditions [20] is the ratio between the energy extracted from the battery during the discharge Δ*E_Load_* [J] and the net energy of the battery Δ*E_Battery output_* [J], which can be expressed as

(10) $$\eta_{Discharge}=\frac{\Delta E_{Load}}{\Delta E_{Battery\;output}}=\frac{\Delta E_{Battery\;output}-\Delta E_{Dischargingloss}}{\Delta E_{Battery\;output}}$$

where Δ*E_Discharging loss_* [J] is the energy loss in the battery during the discharging cycles.

The energy extracted from the battery during the discharge, Δ*E_Load,_* can be determined as

(11) $$\Delta E_{Load}=\int_{t_{0}}^{t}V_{Discharge}I_{Discharge}dt=\int_{SoC(t_{0})}^{SoC(t)}V_{Discharge}C_{n}dSoC\;[J]$$

where *V_Discharge_* [V] is the battery voltage during the discharge process.

Finally, the overall energy efficiency of the charge and discharge cycle [20] is determined as the ratio between Δ*E_Power source_* and Δ*E_Load_*,

(12) $$\eta_{Battery}=\frac{\Delta E_{Load}}{\Delta E_{Power\;source}}$$

### 2.3. Proposed Method to Determine the Energy Balance of the Battery

The efficiencies in (6)–(12) are generally determined for constant charge and discharge rates. However, in practical applications, the charge and discharge rates are not constant. To determine the maximum data transfer per hour (DTR_max_), that is, the number of communication cycles that the IoT device can perform each hour without draining the battery, the energy input and output of the battery must be measured in a real situation. In this case, the energy harvesting unit supplies the load (sensors and communication modules) through the battery, so it must be accomplished

(13) $$E_{Battery\;input}=E_{out\_DC-DC}\eta_{Charge}\;[J]$$

where *E_out_DC–DC_* is the electrical energy at the output of the DC–DC converter, the power source in this case, and *η_Charge_* is given by (6).

The output power delivered by the battery can be calculated as

(14) $$E_{Battery\;output}=\frac{E_{Load}}{\eta_{Discharge}}\;[J]$$

where *E_Load_* is the energy consumed by the IoT device (sensors and communication modules), the load of the analyzed circuit, and *η_Discharge_* is given by (10).

Finally, the energy balance is reached when the energy harvested is equal to the energy consumed by the load

(15) $$E_{Battery\;input}=E_{Battery\;output}$$

Applying the energy balance to the battery for 1 h results in:

(16) $$E_{Battery\;input,1\;h}=E_{Battery\;output,1\;h}=\frac{E_{Load,1\;h}}{\eta_{Discharge}}=\frac{{\mathrm{DTR}}_{\max}(E_{Load,1\;\mathrm{communication}\;\mathrm{cycle}}+E_{sleep})}{\eta_{Discharge}}$$

**Figure 2:**
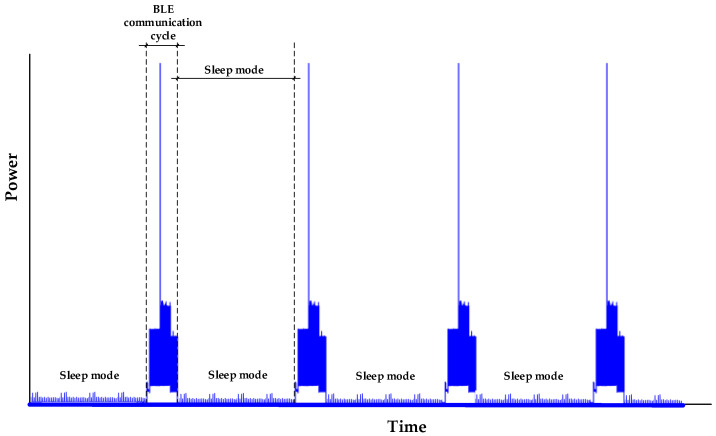
Power consumed by the IoT device (*SmartConnector*) during multiple BLE communication cycles separated by sleep mode periods.

Finally, the maximum data transfer rate DTR_max_ is obtained as

(17) $${\mathrm{DTR}}_{\max}=\frac{E_{Battery\;input,1\;h}}{E_{Load,1\;\mathrm{communication}\;\mathrm{cycle}}+E_{sleep}}\eta_{Discharge}=\frac{E_{out\_DC-DC,1\;h}}{E_{Load,1\;\mathrm{communication}\;\mathrm{cycle}}+E_{sleep}}\eta_{Charge}\eta_{Discharge}$$

where *E_Load,1 communication cycle_* and *E_sleep_* are shown in Figure 2.

## 3. Experimental Setup

This section describes the experimental setup required to determine the energy efficiency of the entire IoT device and to determine the DTR_max_.

### 3.1. Energy Harvesting System

As explained, in the analyzed application, there is a small temperature gradient between the busbar and the environment, so the selection of the most appropriate TEG is critical. Due to the low temperature difference, the TEG generates a very low voltage of a few fractions of a volt, thus requiring a DC–DC converter with a very low input voltage range.

The GM250-157-14-16 TEG from European Thermodynamics (Kibworth, Leicestershire, United Kingdom) was selected due to its ability to handle small temperature gradients. The dimensions of this TEG are 40 mm × 40 mm × 4.1 mm.

The selected DC–DC converter is the LTC3108 from Analog Devices (Wilmington, Massachusetts, USA), which is linked to an ADEH harvesting board based on maximum power point tracking (MPPT) technology and a high efficiency boost converter with an input voltage range of 50–400 mV and an output voltage output range of 2.35–5.0 V.

**Figure 3:**
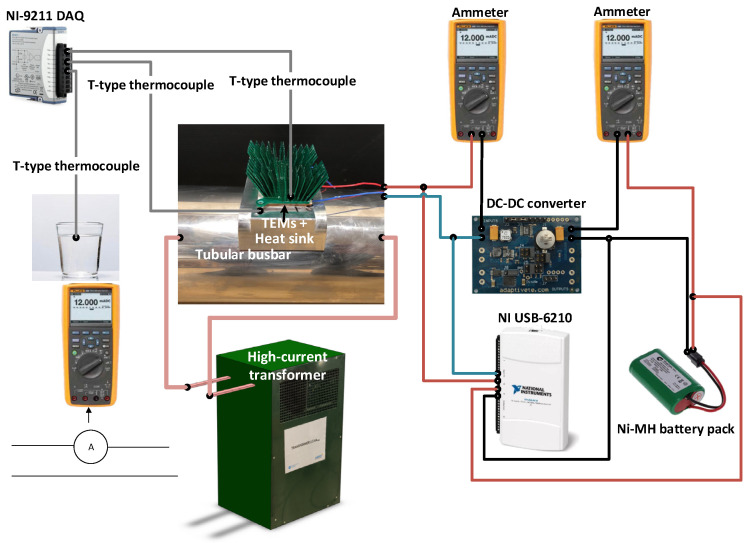
Experimental setup to test the behavior of the energy harvesting system mounted on a tubular busbar.

Figure 3 shows the experimental setup used to test the energy harvesting test, which is composed of a conductor loop. This loop was exposed to heating and cooling cycles. The low impedance loop consists of a stainless-steel tubular busbar with an inner diameter of 120 mm and a wall thickness of 0.4 mm, connected to the output of a high current transformer. The energy harvesting unit includes two TEGs (thermoelectric generators) connected in series and a DC–DC converter. A Ni-MH battery pack composed of two cells in series was also used for power management purposes. In order to test the energy harvesting system in a realistic situation, the TEGs and the DC–DC converter were installed on the top of the tubular busbar, which was exposed to the heat cycle tests, as shown in Figure 3.

Regarding the measurement systems, two Fluke 289 data logger multimeters (Fluke, Everett, Washington, DC, USA) were used in ammeter mode to measure the output currents of the TEGs and the DC–DC converter. Simultaneously, a NI USB-6210 data acquisition system (National Instruments, Austin, TX, USA) was used to acquire the output terminal voltages of the TEGs and DC–DC converter. Three T-type thermocouples together with a NI-9211 temperature measurement system (National Instruments, Austin, TX, USA) were used to measure the environment temperature and the temperatures of the hot and cold sides of the TEGs. A Python code programmed by the authors of this work was used to synchronize all measurement systems.

### 3.2. Energy Storage

Rechargeable Ni-MH batteries are widely used in consumer electronics, such as digital cameras or portable electronic devices [23]. Therefore, we proposed to use a Ni-MH pack of two rechargeable battery cells to store the energy generated by the energy harvesting system (TEG + DC–DC converter) for the IoT device (BM2000C1450AA2S1PATP, GlobTek, Northvale, New Jersey, USA). Table 1 shows the main characteristics of the battery pack analyzed in this work.

As shown in Table 1, the two series connected rechargeable Ni-MH batteries used in this application generate around 2.4 V. Ni-MH batteries were selected because this voltage level is directly compatible with that required by the electronic sensors and the microcontroller that includes an inbuilt BLE module (see Section 3.3), which is between 1.8 V and 3.0 V.

The electronics incorporated in the *SmartConnector* IoT device consume very little power, in the milliwatt range [4]. Therefore, to analyze the behavior of the Ni-MH battery used in this IoT application, the charge and discharge profiles of the batteries analyzed in this paper require very low C rates, the unit to measure the speed at which a battery charges or discharges. For example, a charge cycle at a C rate of *n*^−1^ C means that the battery is charged from 0% to 100% in *n* hours.

**Figure 4:**
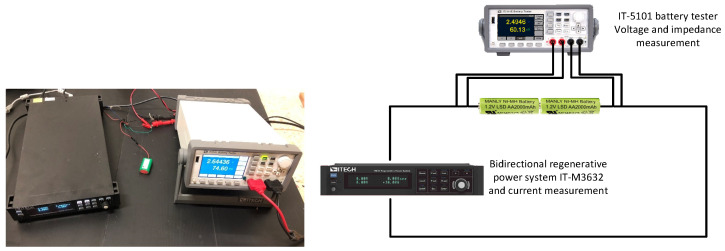
Experimental setup used for battery charge–discharge cycle tests.

Figure 4 shows the experimental setup implemented in this work to analyze the behavior of the rechargeable battery pack. The charge and discharge experiments were performed using a bidirectional regenerative power system (IT-M3632, 800 W, 60 V, 30 A, ITECH, New Taipei City, Taiwan) connected to the two terminals of the battery pack. This instrument measures and records voltage and current with an accuracy of ±0.1% and 0.1% + 0.1% FS, respectively. Simultaneously, a battery tester (IT-5101, ITECH, New Taipei City, Taiwan) was used to measure the voltage and internal impedance of the battery with an accuracy of ±(0.01% + 0.01% FS) and ±(0.4% + 0.05% FS), respectively. The measurements of the voltage and impedance of the battery from the tester were synchronized with a computer using a Python code programmed by the authors of this work.

### 3.3. IoT Device

The IoT device analyzed in this work consists of the energy harvesting system described in Section 3.1, the energy storage unit described in Section 3.2, three sensors, temperature (Pt-1000 sensor, PTFC102T1G0, TE Connectivity, Schaffhausen, Switzerland), voltage drop (AD627 instrumentation amplifier from Analog Devices, Wilmington, MA, USA), and current (DVR5053VA Hall effect sensor, Texas Instruments, Dallas, Texas, USA), as well as a Bluetooth low energy (BLE) communications module (nRF52832 microcontroller from Nordic Semiconductors mounted on Sparkfun breakout board that includes an inbuilt BLE module).

**Figure 5:**
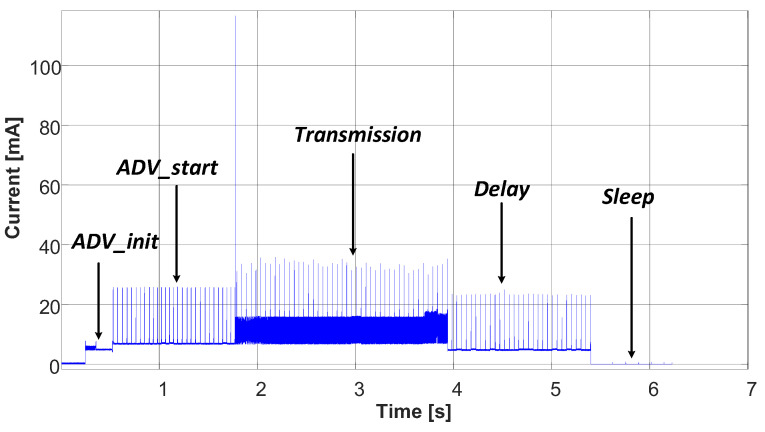
Experimental current versus time profile of the communications cycle.

This section presents two systems to measure the very low energy consumption of the analyzed IoT device. Since this device communicates cyclically with a gateway, the energy consumption has a cyclic profile consisting of five modes, advertising parameter initialization, advertising start, transmission, delay, and sleep, as shown in Figure 5.

**Figure 6:**
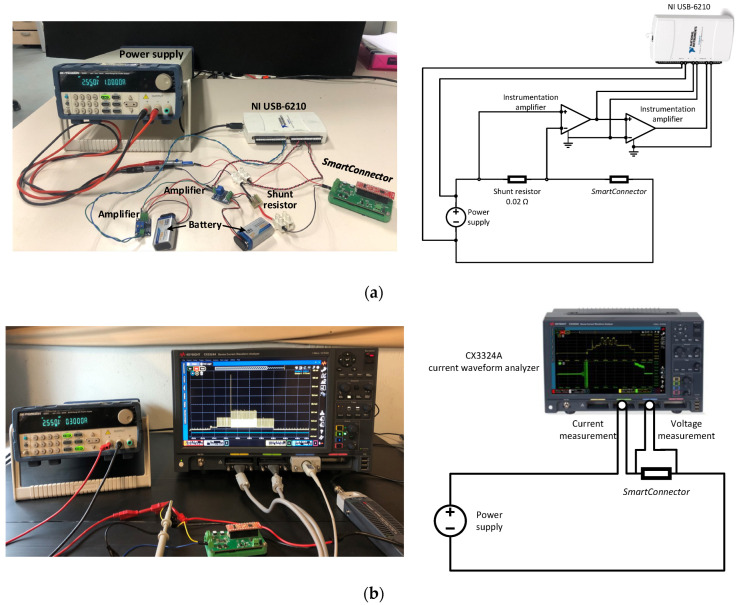
Experimental setup to determine the energy consumption of the IoT device. (**a**) Lab-design system. (**b**) CX3324A current waveform analyzer.

The *SmartConnector* was programmed to enter low power mode during the sleep phase, drawing a few microamps [4]. However, it is very difficult and expensive to acquire current probes for oscilloscopes compatible with this range, being a challenging task to determine the energy consumption of the IoT device. A lab-design data acquisition system was designed for this purpose and assembled, as shown in Figure 6a. It consists of a precision current sense resistor (SR10, 0.02 Ω, ±1%, 1W, Caddock Electronics, Roseburg, OR, USA) and two instrumentation amplifiers (AD620, Analog Devices, Wilmington, MA, USA) connected in cascade that were used to amplify the output voltage. Simultaneously, a wired DAQ module (NI USB-6210, National Instruments, Austin, TX, USA) was connected to the output terminals of the amplifiers and to the power supply to measure both voltages.

To evaluate the accuracy of the measurements made with the lab-design system, a current waveform analyzer (CX3324A, 1 GSa/s, 14/16-bit, 4 Channels, Keysight Technologies, CA, USA) with two current probes (CX1102A Dual Channel, ±12 V, 100 MHz, 40 nA–1 A, Keysight Technologies, Santa Rosa, CA, USA) and one passive voltage probe (N2843A, Keysight Technologies, Santa Rosa, CA, USA) was also used to measure the energy consumed by the *SmartConnector*, which is shown in Figure 6b.

Finally, the energy consumed by the IoT device in one communication cycle, *E_Load, 1 communication cycle_*_,_ can be calculated as

(18) $$E_{Load,\;1\;communication\;cycle}=\int_{t=0}^{t=T}V(t)I(t)dt\;[J]$$

where *V*(*t*) and *I*(*t*) are, respectively, the instantaneous value of the voltage and current measured by the lab-design or CX3324A waveform analyzer, and *T* is the duration of the communication cycle.

## 4. Experimental Results

### 4.1. Energy Harvesting System

This section shows the results of the experimental tests carried out indoors at an ambient temperature of 20 °C to determine the energy generated by the energy harvesting system under different operating conditions.

As already explained, the energy harvesting system is installed on top of a tubular busbar. It consists of two TEGs connected in series, which are connected to a DC–DC converter. The busbar was heated until reaching the steady state temperature by applying currents of different intensities, whose values are summarized in Table 2. After reaching the steady state temperature, the system was cooled to room temperature by natural convection. Therefore, different heating tests were carried out. The powers and efficiencies of the different heat cycle tests summarized in Table 2 are based on Equations (1)–(5). It is noted that Δ*T_Hot-Ambient_* is the temperature gradient between the hot side of the TEG and the ambient, while Δ*T_Hot-Cold_* is the temperature gradient between the hot and cold sides of the TEG.

### 4.2. Battery Efficiency

#### 4.2.1. Experimental Determination of C_n_ and V_OCV_

To determine the efficiency of the battery (charge, discharge and charge-discharge cycles), the rated capacity *C_n_* and the open circuit voltage *V_OCV_* are required, as described in Equations (6)–(12). The following paragraphs explain how they were determined from experimental tests.

According to the IEC 61434 standard [24], the reference current of the test is *I_t_* = *C_n_*/(1 h) [A], and all charge and discharge currents must be expressed as fractions or multiples of *I_t_*. The pack of two rechargeable Ni-MH cells has a rated capacity *C_n_* = 2 Ah (see Table 1), so *I_t_* = 2 A. The end-of-charge voltage per cell was set to 1.75 V, while the end-of-discharge voltage (cut-off voltage) per cell was set to 1.0 V (see Table 1).

**Figure 7:**
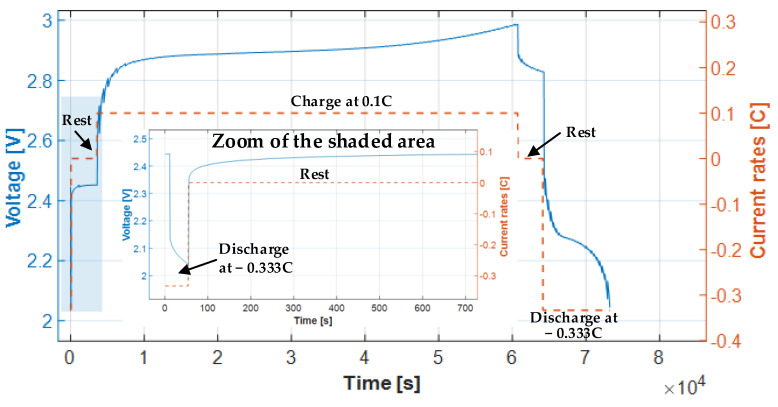
Determination of the rated discharge capacity *C_n_* when the two batteries were discharged at a rate of 0.333*I_t_* = 0.67 A from 3 V to 2 V (end-of-discharge voltage).

The rated capacity *C_n_* [Ah] of the battery pack was calculated according to the procedure described in the IEC 61982 standard [25]. First, the cells were discharged at 25 °C ± 2 °C at a constant current of 0.333*I_t_* (corresponding to 0.67 A) down to 2 V, the end-of-discharge voltage of the two cells specified by the manufacturer. After 1 h of rest, the cells were charged at a constant rate of 0.1*I_t_* (corresponding to 0.20 A) for 16 h at 25 °C ± 2 °C. After another 1 h rest, the batteries were discharged at a constant rate of 0.333*It* (corresponding to 0.67 A) down to 2 V, the end-of-discharge voltage of the two cells. The rated discharge capacity was found to be *C_n_* = 1.75 Ah, as shown in Figure 7.

As shown in Figure 7, the two cells were discharged at a constant rate of 0.333*I_t,_* from an initial voltage of around 2.45 V down to 2.0 V. They were then rested for 1 h, and then charged at a constant current rate of 0.1*I_t_* for 16 h (the charging time specified by the manufacturer, as shown in Table 1) to around 3 V. After another rest of 1 h, the rated discharged capacity *C_n_* was obtained by discharging the cells at a constant rate of 0.333*It* to reach the end-of-discharge voltage (2 V).

(19) $$C_{n}=\int_{t=0}^{t_{EoD}}0.333I_{t}dt=1.75\;[Ah]$$

where *t_EoD_* is the time required to reach the cut-off or end-of-discharge voltage.

It is observed that the obtained value of the rated discharge capacity *C_n_* = 1.75 Ah corresponds to 87.5% of the maximum capacity specified by the manufacturer, which is 2.0 Ah. This is because *C_n_* is highly dependent on how the Ni-MH battery is charged, so it cannot reach the maximum capacity of 2.0 Ah without overcharging due to side reactions [26,27]. Therefore, this study considers the rated capacity *C_n_* of the cells instead of the maximum capacity.

**Figure 8:**
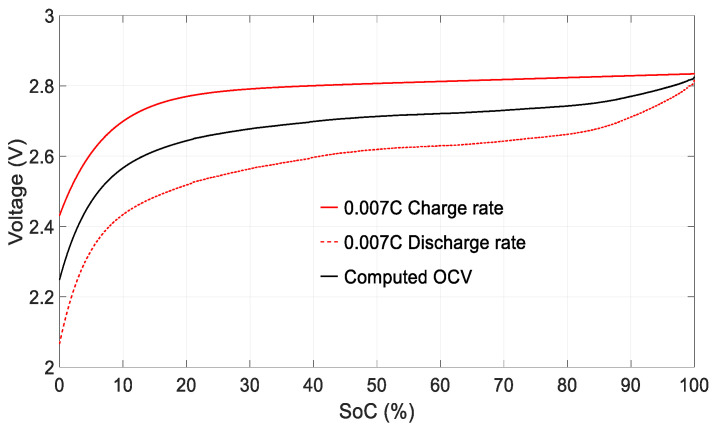
Open circuit voltage *V_OCV_* versus *SoC* obtained by averaging the terminal battery voltage during an entire charge and discharge cycle at 0.007 C.

The open circuit voltage *V_OCV_* of each cell can be obtained from measurements, averaging the charge and discharge curves obtained at very low charge and discharge rates as a function of the *SoC*. In this way, the effects of hysteresis and ohmic resistance are minimized [19,28]. The method developed by Plett [28] was applied to obtain the *V_OCV_* shown in Figure 8. First, the cells were fully charged at 0.05 C. They were then discharged to the end-of-discharge voltage (2 V) at a rate of 0.007 C (0.007 C = 0.007 × 2 A = 0.014 A). Once discharged, they were charged at a rate of 0.007 C until fully charged. Next, the *V_OCV_* curve was obtained by averaging the charge and discharge curves at a rate of 0.007 C, as shown in Figure 8.

The charge and discharge rate was fixed at 0.007 C for two main reasons. First, this discharge rate is similar to the average current consumed by the *SmartConnector*. Second, it is a very low rate, which helps to minimize the influence of hysteresis and Ohmic resistance.

#### 4.2.2. Battery Efficiencies during the Charge, Discharge and Charge-Discharge Cycles

**Figure 9:**
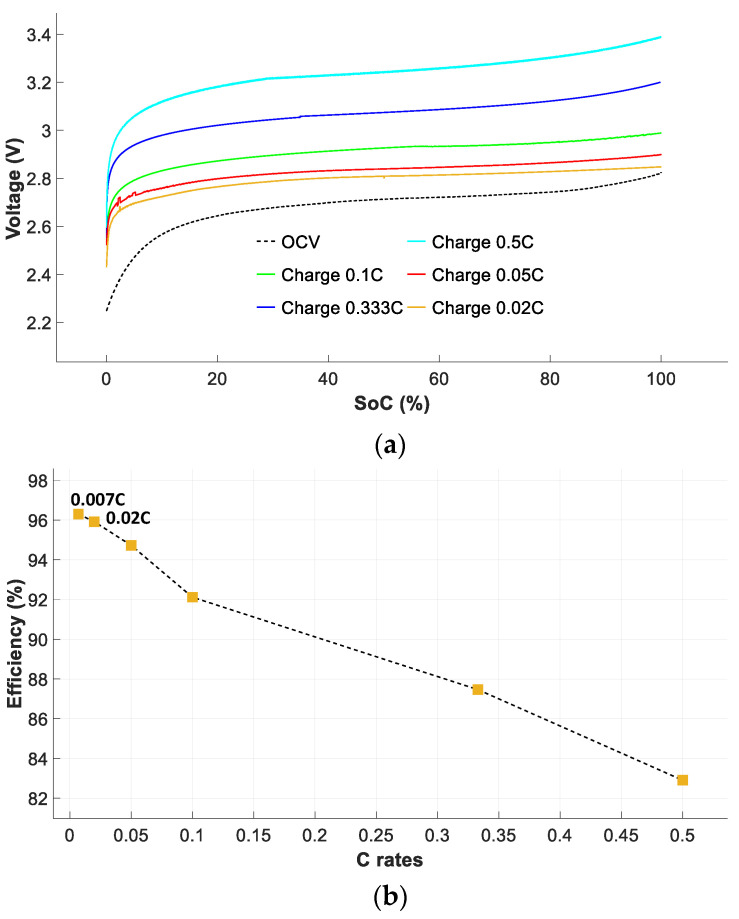
Battery pack performance during the charge cycle. (**a**) Voltage curves as a function of *SoC*. (**b**) Battery charge efficiencies at different current rates.

**Figure 10:**
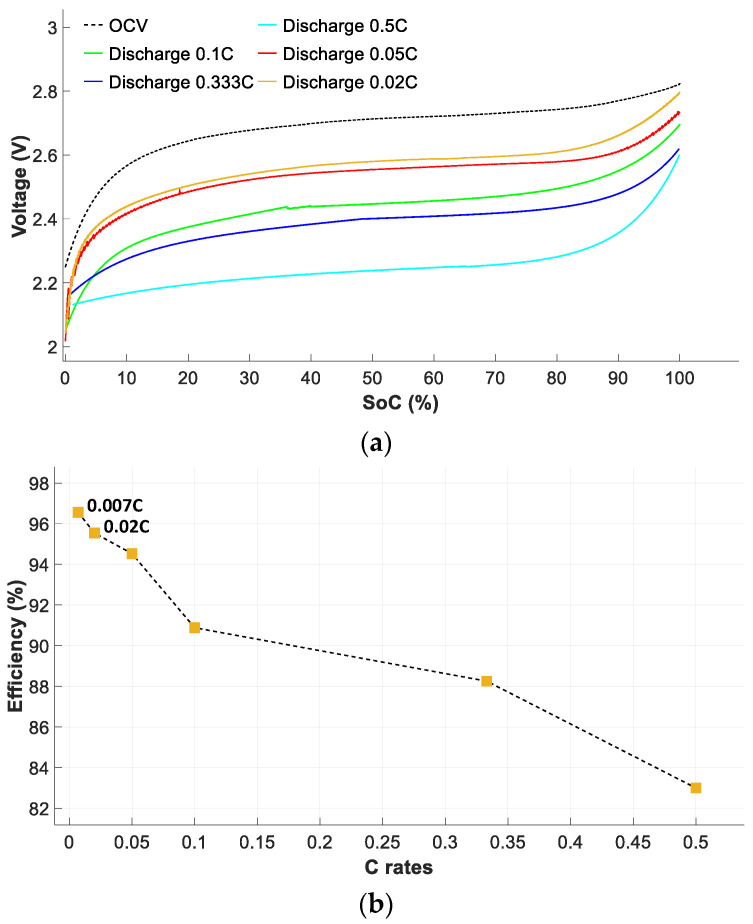
Battery pack performance during the discharge cycle. (**a**) Voltage curves as a function of *SoC*. (**b**) Battery discharge efficiencies at different current rates.

Knowing the rated capacity *C_n_* and the *V_OCV_–SoC* curve, it is possible to determine the efficiency of the battery during the charge and discharge cycles, as well as the overall efficiency from (6), (10) and (12), respectively. To acquire the curves shown in Figure 9 and Figure 10, the cells were first fully charged or discharged at a rate of 0.05 C, and then discharged or charged at different *C* rates to obtain the voltage curves as a function of the *SoC*. Figure 9a shows the battery voltage versus *SoC* during the charge cycle, while Figure 9b shows the charge efficiency versus the C rate.

Figure 9a shows that the terminal voltage behavior of the battery pack is highly dependent on the *SoC* during the charge cycle. At relatively small C rates, such as 0.02 C, 0.05 C, or 0.1 C, the voltage is always below 3 V for *SoC* = 100%. Otherwise, under moderate C rates (0.333 C and 0.5 C), the voltage level rises significantly above 3 V when the cells are fully charged. The charge efficiency *ƞ_Charge_* shown in Figure 9b was calculated according to (6).

Figure 10a shows the battery voltage versus *SoC* during the discharge cycle, while Figure 10b shows the discharge efficiency versus the C rate.

Figure 10a shows that the terminal voltage behavior of the battery pack is highly dependent on the *SoC* during the discharge cycle. According to these results, *ƞ_Discharge_* obviously decreases at high C rates because the cells cannot fully discharge at higher C rates [19].

Figure 10b shows the discharge efficiency *ƞ_Discharge_* versus *SoC*. The *ƞ_Discharge_* characteristic has been calculated according to (19). These results show that *ƞ_Discharge_* also decreases drastically at higher C rates.

**Figure 11:**
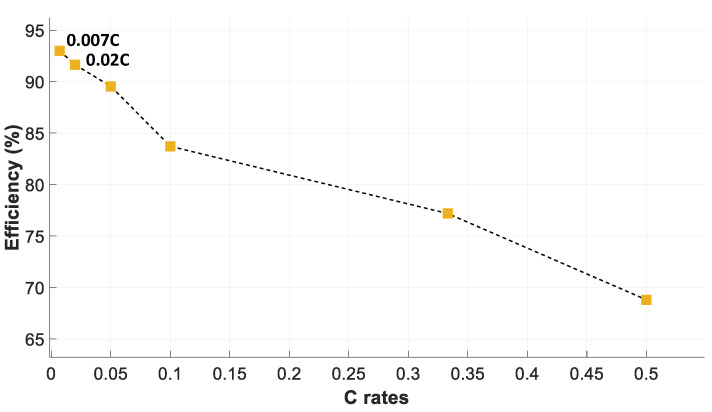
Battery pack efficiency during charge and discharge cycles at different C rates.

Finally, Figure 11 shows the charge and discharge energy efficiency of the battery pack. It is seen that the overall battery efficiency can be as high as 93% at a rate of 0.007 C, thus decreasing at higher C rates. In this case, the overall efficiency during the charge and discharge cycles has been calculated from (12).

The results presented in Figure 11 show that, due to the low level of current generated by the energy harvesting system of the *SmartConnector* and the low current required to supply the sensors and communication modules, the battery will be used efficiently.

### 4.3. Energy Consumption of the IoT Device (Sensors and Communications Module)

This section measures the power and energy consumed by the *SmartConnector*. To this end, the *Smartconnector* was programmed to send data to a nearby gateway every seven seconds.

**Figure 12:**
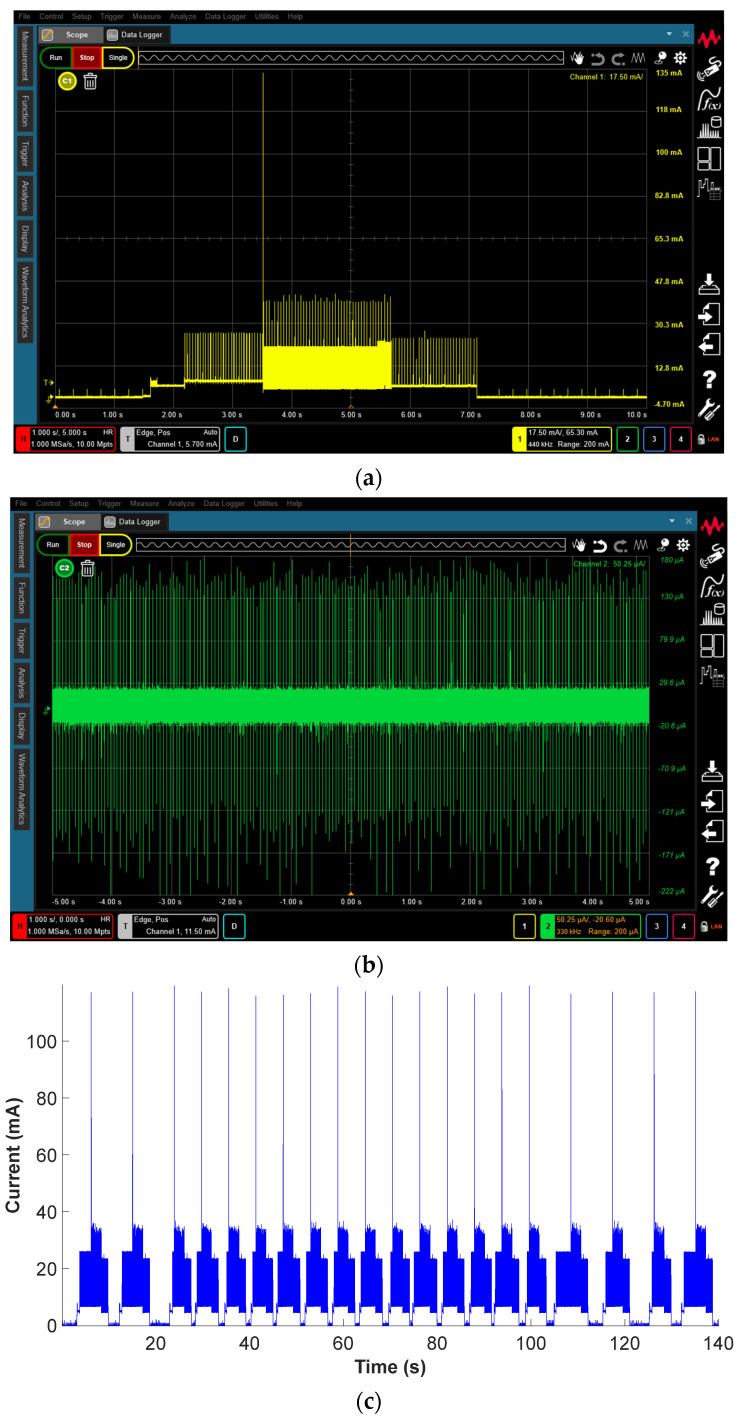
Current consumed by the *SmartConnector*. (**a**) Measured with the CX3324A current waveform analyzer during one entire BLE communication cycle. (**b**) Measured with the CX3324A current waveform analyzer during the sleep mode. (**c**) Measured with the lab-design system during 20 BLE communication and sleep cycles.

Figure 12 shows the current consumed by the *SmartConnector* when supplied with a fixed voltage of 2.55 V. It was measured with the sophisticated CX3324A current waveform analyzer and with the lab-design system.

Table 3 summarizes the results obtained with the two measuring devices in the different phases of power consumption of the BLE communication cycle. These results show that the differences obtained with the CX3324A current waveform analyzer and the lab-design system are very low in all the consumption modes, always less than 5%, which validates the proposed lab-design system.

### 4.4. Energy Balance of the Entire System

This section calculates the energy balance of the *SmartConnector*, from which the DTR_max_ is obtained by applying (17). For this, the energy outputted by the DC–DC converter in 1 h *E_out_DC–DC,1 h_*, the energy consumed by the IoT device during 1 communication cycle *E_Load, 1 communication cycle_*, and the efficiencies of the battery during the charge and discharge cycles, *η_Charge_* and *η_Discharge_*, respectively, were determined.

Table 4 summarizes the energy consumption measured with the lab-design system and the CX3324A current waveform analyzer shown in Figure 12 and Table 3. The results presented in Table 4 have been calculated by averaging the energy consumption of 20 BLE communication cycles, resulting in *E_Load, 1 communication cycle_* = 0.110 J for the lab-design system and *E_Load, 1 communication cycle_* = 0.113 J for the high-performance CX3324A current waveform analyzer, the total energy consumption in one communication cycle.

Table 5 shows the data required to determine the DTR_max_ under different operating conditions defined by different temperature gradients between the environment and the busbar. Since the IoT device consumes a few mA (see Table 4), the battery efficiency has been determined at a current rate of 0.007 C, which corresponds to 14 mA. According to the results presented in Figure 11, the charge and discharge energy efficiency of the battery pack is *ƞ_Charge_ƞ_Discharge_* = 0.93 at a rate of 0.007 C. The maximum data transfer rate DTR_max_ has been determined from (17).

The results presented in Table 5 show a great similitude between the DTR_max_ predicted by the Las-design system and the high performance CX3324A current waveform analyzer. These results also show that even with a temperature difference between the environment and the busbar of only 20 °C, the energy harvesting system allows generating enough energy to sustain a minimum of 5 communications per hour.

## 5. Conclusions

Due to the great expansion of IoT applications, there is a growing interest in developing wireless devices capable of acquiring and transmitting data in transmission lines and electrical substations. This paper has analyzed the behavior of the energy harvesting system, composed of two thermoelectric generators and a DC–DC converter, a rechargeable battery and the sensors and wireless communications modules. Based on experimental tests, an energy balance of the entire IoT device has been carried out, from which the maximum data transfer rate per hour has been determined. The fast switching nature and the small values of the currents of the different communication phases make this measurement challenging. This work has shown that using conventional instrumentation makes it possible to measure the energy involved in the different modes of communication. A detailed energy balance of the battery has also been carried out during charge and discharge cycles, from which the maximum permissible data transfer rate has been determined. It has been shown that, even with small temperature gradients between the environment and the cold side of the thermoelectric generator, it is possible to make several communications per hour. The approach presented here can be generalized to many other smart grid IoT devices.

## Figures and Tables

**Table 1:** Main characteristics of the analyzed battery pack of two cells from GlobTek.

Parameters	Values
Rated capacity, *C_n_*	2000 mAh
Nominal voltage	2.4 V (1.2 V per cell)
Cut-off voltage	2.0 V (1.0 V per cell)
Standard charge current	200 mA (0.1 C), 16 h
Trickle charge current *	60 mA–100 mA (0.03 C–0.05 C)
Rapid charge current	1 A (0.5 C)
Internal impedance	<30 mΩ (upon fully charged)

* Trickle charging means charging a fully charged battery cell at a rate equal to its self-discharge rate, enabling the battery to keep fully charged.

**Table 2:** Power and efficiency of the TEGs and DC–DC converter when the busbar reaches the steady state temperature.

Current (A_rms_)	Δ*T_Hot-Ambient_* (°C)	Δ*T_Hot-Cold_* (°C)	*p_Joule,conductor_* (W/m)	*P_Joule,TEG-area_* (W)	*P_electric,TEG_* (mW)	*P_out_DC–DC_* (mW)	*η_TEG_* (%)	*η_DC–DC_* (%)	*η_TEG+DC/DC_* (%)
136	20.7	3.35	99.5	0.845	1.24	0.21	0.15	17.0	0.025
169	26.1	3.85	155.6	1.321	2.50	0.34	0.19	13.6	0.026
194	31.7	4.46	207.5	1.76	4.47	0.48	0.25	10.7	0.027
226	37.7	5.05	284.0	2.41	7.34	0.66	0.30	9.0	0.027
254	43.7	5.67	362.7	3.08	11.2	0.84	0.36	7.5	0.027

**Table 3:** Current consumption of the IoT device during the different BLE communication modes.

Communication Modes	Supply Voltage [V]	Average Current Measured with the CX3324A Waveform Analyzer [mA]	Average Current Measured with the Lab-Design System [mA]	Difference [%]
Advertising parameters initialization	2.55	5.02	4.97	1.0
Advertising start	2.55	7.44	7.46	0.3
Transmission	2.55	11.42	11.43	0.1
Delay	2.55	4.90	4.89	0.2
Sleep	2.55	0.00510	0.00535	4.9

**Table 4:** Energy consumption of the IoT device in one BLE communication cycle.

	Communication Modes	Voltage [V]	Time [s]	Average Current [mA]	Energy Consumption of Each Mode [J]
Lab-design	Advert. param. initialization	2.55	0.41	4.97	0.005
	Advertising start	2.55	1.18	7.46	0.023
	Transmission	2.55	2.17	11.43	0.064
	Delay	2.55	1.45	4.89	0.018
	Total cycle BLE consumption *	2.55	5.21	8.20	0.110
	Sleep	2.55	1.00	5.35·× 10^−3^	13.64 × 10^−6^
CX3324A	Advert. param. initialization	2.55	0.58	5.02	0.007
	Advertising start	2.55	1.32	7.44	0.025
	Transmission	2.55	2.17	11.42	0.063
	Delay	2.55	1.45	4.90	0.018
	Total cycle BLE consumption *	2.55	5.52	8.10	0.113
	Sleep	2.55	1.00	5.10 × 10^−3^	13.01 × 10^−6^

* The energy consumed in one BLE communication cycle is the sum of the consumptions in each mode (advertising initialization phase, advertising start phase, transmission phase and delay).

**Table 5:** Determination of the maximum data transfer rate for the IoT device.

	Δ*T_Busbar_ambient_* [°C]	*P_out_DC–DC_* [mW]	*E_out_DC–DC, 1 h_* [J]	Baterry Efficiency *ƞ_Charge_ƞ_Discharge_*	DTR_max_ [Communications/h]
Lab-Design	20.7	0.21	0.756	0.93	6 *^a^*
	26.1	0.34	1.224	0.93	10 *^a^*
	31.7	0.48	1.728	0.93	14 *^a^*
	37.7	0.66	2.376	0.93	19 *^a^*
	43.7	0.84	3.024	0.93	25 *^a^*
CX3324A	20.7	0.21	0.756	0.93	5 *^b^*
	26.1	0.34	1.224	0.93	9 *^b^*
	31.7	0.48	1.728	0.93	13 *^b^*
	37.7	0.66	2.376	0.93	19 *^b^*
	43.7	0.84	3.024	0.93	24 *^b^*

*^a^* DTR_max_ calculations assume *E_Load,1 communication cycle_* = 0.110 J and *E_sleep_* = 13.64 × 10^−6^ J/s. *^b^* DTR_max_ calculations assume *E_Load,1 communication cycle_* = 0.113 J and *E_sleep_* = 13.01 × 10^−6^ J/s.

## Data Availability

Not applicable.
